# COVID-19 vaccine booster hesitancy (VBH) of healthcare professionals and students in Poland: Cross-sectional survey-based study

**DOI:** 10.3389/fpubh.2022.938067

**Published:** 2022-07-25

**Authors:** Arkadiusz Dziedzic, Julien Issa, Salman Hussain, Marta Tanasiewicz, Robert Wojtyczka, Robert Kubina, Marta Dyszkiewicz Konwinska, Abanoub Riad

**Affiliations:** ^1^Department of Conservative Dentistry with Endodontics, Medical University of Silesia, Katowice, Poland; ^2^Department of Diagnostics, Poznań University of Medical Sciences, Poznań, Poland; ^3^Doctoral School, Poznań University of Medical Sciences, Poznań, Poland; ^4^Czech National Centre for Evidence-Based Healthcare and Knowledge Translation (Cochrane Czech Republic, Czech EBHC: JBI Centre of Excellence, Masaryk University GRADE Centre), Faculty of Medicine, Masaryk University, Brno, Czechia; ^5^Department of Microbiology and Virology, Medical University of Silesia, Sosnowiec, Poland; ^6^Department of Pathology, Medical University of Silesia, Sosnowiec, Poland; ^7^Department of Anatomy, Poznań University of Medical Sciences, Poznań, Poland; ^8^Department of Public Health, Faculty of Medicine, Masaryk University, Brno, Czechia

**Keywords:** cross-sectional studies, COVID-19 vaccines, decision making, healthcare professionals, vaccination hesitancy, Poland

## Abstract

Since healthcare professionals (HCPs) play a critical role in shaping their local communities' attitudes toward vaccines, HCPs' beliefs and attitudes toward vaccination are of vital importance for primary prevention strategies. The present study was designed as a cross-sectional survey-based study utilizing a self-administered questionnaire to collect data about COVID-19 vaccine booster hesitancy (VBH) among Polish HCPs and students of medical universities (MUSs). Out of the 443 included participants, 76.3% were females, 52.6% were HCPs, 31.8% were previously infected by SARS-CoV-2, and 69.3% had already received COVID-19 vaccine booster doses (VBD). Overall, 74.5% of the participants were willing to receive COVID-19 VBD, while 7.9 and 17.6% exhibited their hesitance and rejection, respectively. The most commonly found promoter for acceptance was protection of one's health (95.2%), followed by protection of family's health (81.8%) and protection of community's health (63.3%). Inferential statistics did not show a significant association between COVID-19 VBH and demographic variables, e.g., age and gender; however, the participants who had been previously infected by SARS-CoV-2 were significantly more inclined to reject the VBD. Protection from severe infection, community transmission, good safety profile, and favorable risk-benefit ratio were the significant determinants of the COVID-19 VBD acceptance and uptake. Fear of post-vaccination side effects was one of the key barriers for accepting COVID-19 VBD, which is consistent with the pre-existing literature. Public health campaigns need to highlight the postulated benefits of vaccines and the expected harms of skipping VBD.

## Introduction

Over the last 2 years, it became evident that coronavirus disease 2019 (COVID-19) transmission chains can be interrupted by herd immunity achieved either by massive vaccination of the community or natural infection (1, 2). Besides the ethical questions about building herd immunity by infection, cost/benefit analysis of this strategy had never been favorable because the burden of casualties was unpredictable (2). For this reason, achieving herd immunity by vaccination was more convincing and reliable.

Since the start of COVID-19 mass vaccination campaigns in December 2020, about 59.3% of the world population has been fully vaccinated (3). As defined by the U.S. Centers for Disease Control and Prevention (CDC), a fully vaccinated person is an individual who is “2 weeks after receiving all recommended doses in the primary series of their COVID-19 vaccination” (4).

Alongside the increase of fully vaccinated individuals toward achieving herd immunity, a decline in the humoral immunity after 6 months of vaccination with the second dose has been reported leading to a new rise of COVID-19 infections (5, 6). Additionally, several COVID-19 variants have been reported since the beginning of the pandemic where only five are classified as variants of concern (VOC) according to their effect on the pandemic situation; Alpha (B.1.1.7), Beta (B.1.351), Gamma (P.1), Delta (B.1.617.2), and Omicron (B.1.1.529) (7). Consequently, the VOCs affected the incidence of COVID-19 infections through rapid dissemination of the infection leading to hospitalization and mortality. Based on the aforementioned obstacles that restrict the process of attaining herd immunity, the mass vaccination campaign needs to continue side by side with the booster or third dose vaccination as a mediator in increasing the immoral immunity and enhancing the vaccine effectiveness (8).

As of September 2021, booster dose vaccination campaigns have been initiated in Poland (9). Despite the type of primary vaccination, the first to receive the booster does were health care professionals (HCPs) that are at risk of COVID-19 infection, together with the individuals aged 50 years old and above that are fully vaccinated for at least 6 months (9). Subsequently, in December 2021 all people aged from 18 to 49 were able to get vaccinated with the booster dose (10). Reportedly, on April 20, 2022, The Polish Ministry of Health announced the launch of the second booster dose vaccination campaign for people aged 80 years old and above who have received the first booster dose for at least 150 days also the immunocompromised individuals from the age of 12 years old were allowed to take the second booster dose if needed (11). Regardless of the efforts promoting third dose vaccination, only 51.8% of the fully vaccinated Poles have taken the first booster dose (12). A study by Rzymski et al. (13) reported a significant level of hesitancy for receiving the COVID-19 vaccine booster dose among the Polish community; furthermore, another study by Babicki and Mastalerz-Migas (14) reported a low level of booster dose acceptance among Poles. The previously experienced vaccine side effects and the booster dose safety and effectiveness were the primary reasons for the hesitancy toward COVID-19 third dose vaccination (13). Therefore, the present study was carried out to specifically target Polish HCPs and evaluate their views and attitudes toward COVID-19 vaccine booster doses (VBD).

The World Health Organization (WHO) defines vaccine hesitancy as “delay in acceptance or refusal of vaccines despite availability of vaccine services” (15). The risk factors of vaccine hesitancy can be classified according to the 3-C model of the WHO-Strategic Advisory Group of Experts on Immunization (SAGE), including complacency, convenience, and confidence (16). The three core elements of vaccine hesitancy are usually mediated by individuals' vaccine-related knowledge and health literacy levels (17–19). The health-related beliefs and attitudes of HCPs play a significant role in primary prevention and health promotion as they are broadly perceived as role models and credible sources of health information (19, 20). Therefore, COVID-19 booster dose hesitancy among HCPs may negatively impact public confidence in booster doses (21).

The overarching goal of this study was to evaluate COVID-19 vaccine booster hesitancy (VBH) among HCPs and medical universities students (MUSs) in Poland. The primary objective was to estimate the prevalence of COVID-19 VBH among Polish HCPs and MUSc, while the secondary objectives were: (i) to evaluate the demographic, anamnestic, and psychosocial drivers of COVID-19 VBD-related acceptance and (ii) to assess the correlation between COVID-19 VBD-related acceptance and actual VBD uptake among the target population.

## Materials and methods

### Design

The present work had been designed as an analytical cross-sectional survey-based study that was carried out between December 2021 and January 2022. A self-administered questionnaire (SAQ) was used for the purpose of data collection after being digitally designed using KoBoToolbox (Harvard Humanitarian Initiative, Cambridge, MA, USA, 2021) (22). The study had been designed and reported in full compliance with the Strengthening the Reporting of Observational Studies in Epidemiology (STROBE) guidelines for cross-sectional studies (23).

### Participants

The target population of this study were HCPs and MUSc in Poland. The exclusion criteria were: (i) not working as a HCP or studying at a medical university, (ii) providing insufficient demographic information, and (iii) not providing their informed consent *a priori*. The participation in this study was completely voluntary, the participants received no financial rewards or any other means of incentives to take part in this study. The participants' interest, especially the students, in participating in this study was not coerced by any means of threats. The participants' identity was kept anonymous in order to control the Hawthorne's effect and information bias.

A non-random sampling strategy was used for data collection through convenience recruitment. The participants were invited to this study through multiple channels in two major academic centers, Katowice and Poznan. A uniform resource locator (URL) and quick response (QR) code for the questionnaire were sent to the potential participants as they were able to download it from the project promoting sources, such as Medical Universities websites, scientific societies and professional regulatory bodies.

The pragmatic sample size required for this study was computed using Epi-Info™ version 7.2.5 (CDC. Atlanta, GA, USA, 2021), specifically through the module of “Population Survey” (24, 25). Following the assumptions of 5% as an error margin, 97% as a confidence level, 71% as an expected outcome frequency which was based on a recent study for Polish adults, and 10% as a postulated proportion of faulty responses due to careless/insufficient efforts, the required sample was 427 responses (13).

A total of 456 responses were received from the potential participants, 13 of which were excluded due to insufficiency of demographic information that were crucial to their classification and subsequent analysis.

### Instrument

The SAQ that was used in this study had been used in previous studies concerned with evaluating COVID-19 VBH in Czechia and Germany (21, 26). The psychometric validation process comprised of content validity evaluation and test re-test reliability which showed that this SAQ had substantial reliability denoted by a mean Cohen's kappa coefficient of 0.80 ± 0.19 (IQR: 0.60–1.00) (21).

The SAQ was consisted of 17 items that were divided into four basic sections; (i) demographic information: gender, age, profession, and geographic region, (ii) COVID-19-related anamnesis: prior infection, its onset and severity, vaccination history, number of doses, and post-vaccination hospitalization and medical care, (iii) willingness to receive COVID-19 VBD evaluated by a 5-point Likert scale ranging from “Totally Disagree = 1” to “Totally Agree = 5,” and (iv) psychosocial drivers of COVID-19 VBH; e.g., protection against severe infection and community transmission.

The attitudes toward COVID-19 VBD were stratified into three levels based on the responses to the 5-point Likert scale: “VBD Rejection” group included those who responded “Totally Disagree” and “Disagree,” “VBD Hesitancy” group included those who responded “Not Sure,” and “VBD Acceptance” group included those who responded “Agree” and “Totally Agree.” To facilitate the subsequent analyses, the participants who received the third dose of the vaccine were denoted as “Triple Vaccinated.”

### Ethics

The proposed study protocol had been reviewed and approved by the Ethics Committee of the Medical University of Silesia on 20 July 2021 (PCN/CBN/0022/KB/161/21). The Declaration of Helsinki for research involving human subjects and the European Union (EU) General Data Protection Regulation (GDPR) governed the process of data collection, storing, and handling (27, 28). All the participants provided their informed consent digitally prior to their participation, and no information or responses were collected before that point. The study participants were allowed to leave the study at any moment without the need to justify their decision. No identifying personal data, e.g., email or telephone number was collected from the participants; therefore, retrospective identification of the participants was not possible.

### Analyses

All descriptive and inferential statistical tests were performed using the Statistical Package for the Social Sciences (SPSS) version 28.0 (SPSS Inc. Chicago, IL, USA, 2021) except for regression analyses that were performed using the R-based open software “Jamovi” (29, 30). Shapiro Wilk test was used to evaluate the distribution of numerical variables with a significance level (*Sig*.) of 5%. Frequencies (*n*) and percentages (%) were used to evaluate present the categorical and ordinal variables such as gender, pregnancy, vaccination status, attitudes toward COVID-19 VBD, and psychosocial drivers, while means, standard deviations and interquartile ranges (μ ± SD “IQR”) were used for numerical variables, e.g., age. Subsequently, inferential tests such as Chi-squared test (χ^2^), Fisher's exact test, and Mann-Whitney (U) test were used to evaluate the association between dependent and independent variables. Bivariate correlation using the non-parametric test of Spearman's rank was performed between COVID-19 VBD attitudes and actual uptake. Finally, the multivariable logistic regression was used to estimate the adjusted odds ratio (*AOR*) of various psychosocial drivers for COVID-19 VBD acceptance and actual uptake. The regression analysis was adjusted for the demographic and anamnestic variables that were found to be significant in the univariate analysis. All inferential tests were performed with a confidence level (*CI*) of 95% and a significance level (*Sig*.) of 5%.

## Results

### Demographic characteristics

A total of 443 participants were included in this study, out of which 233 (52.6%) were HCPs and 210 (47.4%) were MUSc. In general, females were the vast majority (76.3%), followed by males (22.8%) and diverse-gender (0.9%) participants without significant differences between professionals' and students' groups. Out of the 338 participating females, only 7 (2.1%) were pregnant and they all belonged to the professionals' group. The mean age of the sample was 31.1 ± 11.4 with a statistically significant difference (*Sig*. <0.001) between professionals (38.8 ± 10.9) and students (22.6 ± 2.3) (Table 1).

**Table 1:** Demographic characteristics of polish healthcare professionals and students responding to COVID-19 VBH survey, December 2021–January 2022 (*n* = 443).

**Variable**	**Outcome**	**Professionals** **(*****n*** = **233)**	**Students** **(*****n*** = **210)**	**Total** **(*****n*** = **443)**	***Sig***.
Gender	Female^†^	175 (75.1%)	163 (77.6%)	338 (76.3%)	*Reference*
	Male	55 (23.6%)	46 (21.9%)	101 (22.8%)	0.636
	Diverse-gender	3 (1.3%)	1 (0.5%)	4 (0.9%)	0.376
Pregnancy^†^	Yes	7 (4%)	0 (0%)	7 (2.1%)	0.015
	No	168 (96%)	163 (100%)	331 (97.9%)	
Age	μ ±*SD* (IQR)	38.8 ± 10.9 (31–45)	22.6 ± 2.3 (21–24)	31.1 ± 11.4 (23–36.3)	<0.001

*Logistic regression, Fisher's exact test, and Mann-Whitney test (U) had been used with a significance level (Sig.) <0.05*.

† * Refers to female participants*.

*Bold values - statistically significant with p <0.05*.

The most participating region was Silesian Voivodeship (54.4%), followed by the Greater Poland Voivodeship (28.9%), and the Lesser Poland Voivodeship (8.1%).

### Anamnestic characteristics

Nearly one-third (31.8%) of the participants reported being infected previously with COVID-19, and the vast majority of them were infected before receiving the first dose (73%), followed by those who were infected after the second dose (22.7%), and those who were infected between the doses (4.3%). According to the Australian guidelines for clinical classification of COVID-19 patients, most of our participants experienced mild infection (66%), followed by moderate (29.1%), asymptomatic (2.8%), and severe infection (2.1%). There was no significant difference between professionals' and students' groups in terms of COVID-19 infection-related anamnesis.

The vast majority of the participants (93.7%) reported receiving at least one dose of COVID-19 vaccines without a significant difference between professionals and students. As expected, the most common vaccine type was Pfizer-BioNTech (78.3%) which was significantly (*Sig*. <0.001) more common among professionals (89.3%) than students (66.7%). AstraZeneca-Oxford was the second most common vaccine type (13%) and it was significantly (*Sig*. <0.001) more common among students (22.9%) than professionals (3.7%). To a limited extent, Moderna and Janssen vaccines were received by 4.8 and 3.9% of the participants. Most of the participants were triple vaccinated (74%), with a significant difference (*Sig*. <0.001) between professionals (79%) and students (68.7%). Only 4.3% of the whole sample received a single vaccine dose, and 3.4 and 4.3% reported post-vaccination hospitalization and seeking medical care (Table 2).

**Table 2:** Anamnestic characteristics of polish healthcare professionals and students responding to COVID-19 VBH survey, December 2021–January 2022 (*n* = 443).

**Variable**	**Outcome**	**Professionals** **(*****n*** = **233)**	**Students** **(*****n*** = **210)**	**Total** **(*****n*** = **443)**	***Sig***.
Prior COVID-19 infection	Yes^†^	72 (30.9%)	69 (32.9%)	141 (31.8%)	0.659
	No	161 (69.1%)	141 (67.1%)	302 (68.2%)	
Onset^†^	Before 1st dose	50 (69.4%)	53 (76.8%)	103 (73%)	*Reference*
	Between 1/2 doses	2 (2.8%)	4 (5.8%)	6 (4.3%)	0.475
	After 2nd dose	20 (27.8%)	12 (17.4%)	32 (22.7%)	0.170
Severity^†^	Asymptomatic	2 (2.8%)	2 (2.9%)	4 (2.8%)	*Reference*
	Mild	49 (68.1%)	44 (63.8%)	93 (66%)	0.916
	Moderate	19 (26.4%)	22 (31.9%)	41 (29.1%)	0.889
	Severe	2 (2.8%)	1 (1.4%)	3 (2.1%)	0.661
COVID-19 vaccination	Yes ^‡^	214 (91.8%)	201 (95.7%)	415 (93.7%)	0.095
	No	19 (8.2%)	9 (4.3%)	28 (6.3%)	
Vaccine type^‡^	Pfizer-BioNTech	191 (89.3%)	134 (66.7%)	325 (78.3%)	<0.001
	Moderna	9 (4.2%)	11 (5.5%)	20 (4.8%)	0.547
	AstraZeneca-Oxford	8 (3.7%)	46 (22.9%)	54 (13%)	<0.001
	Janssen	6 (2.8%)	10 (5%)	16 (3.9%)	0.251
Number of doses^‡^	One dose	8 (3.7%)	10 (5%)	18 (4.3%)	0.536
	Two doses	37 (17.3%)	53 (26.4%)	90 (21.7%)	0.025
	Three doses	169 (79%)	138 (68.7%)	307 (74%)	0.017
Booster recipient	Yes	169 (72.5%)	138 (65.7%)	307 (69.3%)	0.120
	No	64 (27.5%)	72 (34.3%)	136 (30.7%)	
Hospital admission^‡^	Yes	11 (5.1%)	3 (1.5%)	14 (3.4%)	0.040
	No	203 (94.9%)	198 (98.5%)	401 (96.6%)	
Medical care^‡^	Yes	11 (5.1%)	7 (3.5%)	18 (4.3%)	0.407
	No	203 (94.9%)	194 (96.5%)	397 (95.7%)	

*Logistic regression and Chi-squared test (χ^2^) had been used with a significance level (Sig.) <0.05*.

† * Refers to the previously infected participants*.

‡ * Refers to the previously vaccinated participants*.

*Bold values - statistically significant with p <0.05*.

### COVID-19 vaccine booster dose (VBD)-related attitudes

Overall, almost three-quarters (74.5%) of the participants indicated their acceptance to receive COVID-19 VBD, while 17.6% indicated their rejection, and 7.9% were hesitant. No significant difference between professionals and students in terms of VBD-related attitudes. The triple vaccinated individuals had a significantly (*Sig*. <0.001) higher level of VBD acceptance (87.9 vs. 44.1%) and a significantly (*Sig*. <0.001) lower level of VBD rejection (8.1 vs. 39%) compared with their counterparts who did not receive the third dose, respectively.

When asked about their reasons to accept COVID-19 VBD, the most commonly reported promoted was protection of one's own health (96.3%), followed by protection of family's health (82.5%), and protection of community's health (65%). On the other hand, work or study place endorsement (5%) and avoidance of frequent testing (20%) were the least reported promoters. The students were significantly more inclined to indicate testing avoidance (20 vs. 11.2%) and having easier social life with less restrictions (58.8 vs. 43.5%) than the professionals, respectively (Table 3).

**Table 3:** Attitudes toward COVID-19 VBD of polish healthcare professionals and students responding to COVID-19 VBH survey, December 2021–January 2022 (*n* = 443).

**Variable**	**Outcome**	**Employment**			**Triple vaccinated**			**Total** **(*****n*** = **443)**
		**Professionals** **(*****n*** = **233)**	**Students** **(*****n*** = **210)**	***Sig***.	**Yes** **(*****n*** = **307)**	**No** **(*****n*** = **136)**	***Sig***.	
Attitudes	Rejection	45 (19.3%)	33 (15.7%)	0.321	25 (8.1%)	53 (39%)	<0.001	78 (17.6%)
	Hesitancy	18 (7.7%)	17 (8.1%)	0.885	12 (3.9%)	23 (16.9%)	<0.001	35 (7.9%)
	Acceptance^†^	170 (73%)	160 (76.2%)	0.436	270 (87.9%)	60 (44.1%)	<0.001	330 (74.5%)
Promoter^†^	Self-protection	160 (94.1%)	154 (96.3%)	0.367	256 (94.8%)	58 (96.7%)	0.746	314 (95.2%)
	Family's health	138 (81.2%)	132 (82.5%)	0.755	220 (81.5%)	50 (83.3%)	0.737	270 (81.8%)
	Patient/colleague	89 (52.4%)	93 (58.1%)	0.292	148 (54.8%)	34 (56.7%)	0.794	182 (55.2%)
	Community's health	105 (61.8%)	104 (65%)	0.542	167 (61.9%)	42 (70%)	0.236	209 (63.3%)
	Testing avoidance	19 (11.2%)	32 (20%)	0.027	42 (15.6%)	9 (15%)	0.914	51 (15.5%)
	Easier social life	74 (43.5%)	94 (58.8%)	0.006	138 (51.1%)	30 (50%)	0.876	168 (50.9%)
	Work/study place	4 (2.4%)	8 (5%)	0.199	9 (3.3%)	3 (5%)	0.463	12 (3.6%)

*Chi-squared test (χ^2^) and Fisher's-exact test had been used with a significance level (Sig.) <0.05*.

† * Refers to the vaccine-accepting group*.

*Bold values - statistically significant with p <0.05*.

### Psychosocial drivers of COVID-19 vaccine booster hesitancy (VBH)

More than three-quarters (76.1%) of the participants agreed with the notion that VBD were capable of preventing severe infection, with a significant difference (*Sig*. <0.001) between triple vaccinated participants (87.9%) and their counterparts (49.3%) and with no significant difference (*Sig*. = 0.495) between professionals (76.4%) and students (75.7%). Similarly, the notion that VBD were able to prevent symptomatic infection was significantly (*Sig*. <0.001) more accepted by the triple vaccinated participants (73.6%) than their counterparts (38.2%). Moreover, the notion that VBD were able to prevent community transmission was significantly (*Sig*. <0.001) more accepted by the triple vaccinated participants (65.1%) than their counterparts (27.9%).

Interestingly, 68.4% of the participants did not agree to postpone receiving of their VBD until they found convincing evidence that the VBD would control the emerging variants. While there was no statistically significant (*Sig*. = 0.407) difference between professionals (72.1%) and students (64.3%) in the notion of variants control, the triple vaccinated participants (80.8%) were significantly (*Sig*. <0.001) more inclined to disagree with this notion compared with their counterparts (40.4%).

**Figure 1:**
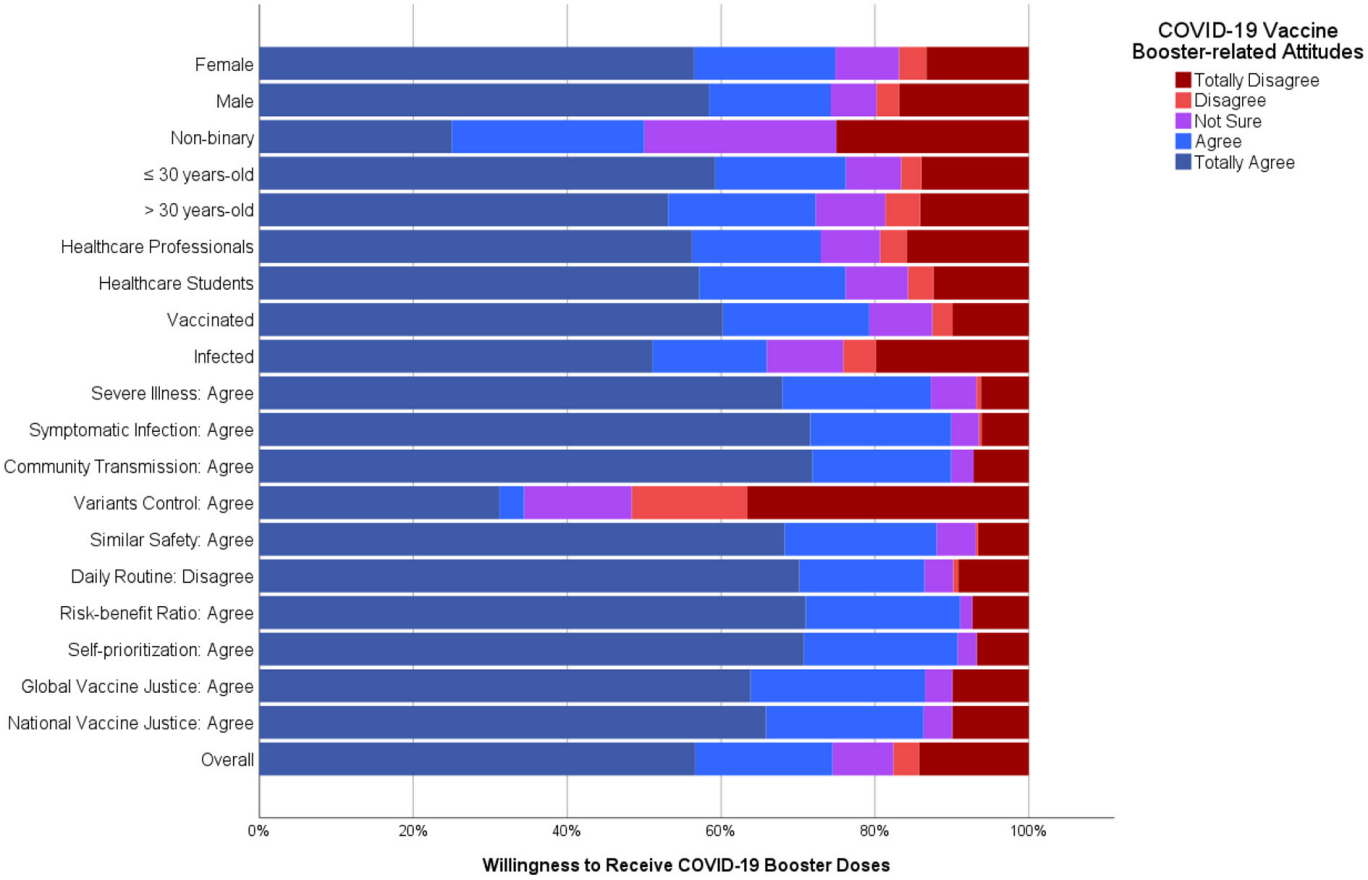
Determinants of COVID-19 vaccine booster-related attitudes of polish healthcare professionals and students responding to COVID-19 VBH survey, December 2021–January 2022 (*n* = 443).

About three-quarters (75.4%) of the participants agreed with the notion that VBD would be as safe as the primer doses, with a significant difference (*Sig*. <0.001) between triple vaccinated participants (86.3%) and their counterparts (50.7%) and with no significant difference (*Sig*. = 0.280) between professionals (73.8%) and students (77.1%). Almost two-thirds (66.6%) of the participants disagreed with the notion the VBD would cause severe side effects interfering with their daily routine, with a significant difference (*Sig*. <0.001) between triple vaccinated participants (76.9%) and their counterparts (43.4%) and with no significant difference (*Sig*. = 0.777) between professionals (65.7%) and students (67.6%) (Figure 1).

A large proportion of the participants agreed that the benefits of VBD would outweigh their risks (70.9%) and that they should be prioritized to receive the VBD based on their occupational risk (73.5%). However, the differences between professionals and students were not statistically significant for both notions, the triple vaccinated participants had significantly higher agreement levels with both of them (84.4 and 88.2%, respectively) compared with their counterparts (40.4 and 40.4%, respectively).

The positions of our participants from the ethical dilemmas of vaccine justice either globally or nationally was almost equally distributed between agreement and disagreement, without significant differences between professionals and students (Table 4).

**Table 4:** Determinants of COVID-19 VBH among polish healthcare professionals and students responding to COVID-19 VBH survey, December 2021–January 2022 (*n* = 443).

**Variable**	**Outcome**	**Employment**			**Triple vaccinated**			**Total** **(*****n*** = **443)**
		**Professionals** **(*****n*** = **233)**	**Students** **(*****n*** = **210)**	***Sig***.	**Yes** **(*****n*** = **307)**	**No** **(*****n*** = **136)**	***Sig***.	
Severe infection	Agreement	178 (76.4%)	159 (75.7%)	0.495	270 (87.9%)	67 (49.3%)	<0.001	337 (76.1%)
	Disagreement	39 (16.7%)	29 (13.8%)		19 (6.2%)	49 (36%)		68 (15.3%)
Symptomatic infection	Agreement	144 (61.8%)	134 (63.8%)	0.324	226 (73.6%)	52 (38.2%)	<0.001	278 (62.8%)
	Disagreement	52 (22.3%)	38 (18.1%)		30 (9.8%)	60 (44.1%)		90 (20.3%)
Community transmission	Agreement	130 (55.8%)	108 (51.4%)	0.687	200 (65.1%)	38 (27.9%)	<0.001	238 (53.7%)
	Disagreement	57 (24.5%)	52 (24.8%)		44 (14.3%)	65 (47.8%)		109 (24.6%)
Variants control	Agreement	47 (20.2%)	46 (21.9%)	0.407	29 (9.4%)	64 (47.1%)	<0.001	93 (21%)
	Disagreement	168 (72.1%)	135 (64.3%)		248 (80.8%)	55 (40.4%)		303 (68.4%)
Equal safety	Agreement	172 (73.8%)	162 (77.1%)	0.280	265 (86.3%)	69 (50.7%)	<0.001	334 (75.4%)
	Disagreement	36 (15.5%)	25 (11.9%)		19 (6.2%)	42 (30.9%)		61 (13.8%)
Daily routine	Agreement	36 (15.5%)	36 (17.1%)	0.777	37 (12.1%)	35 (25.7%)	<0.001	72 (16.3%)
	Disagreement	153 (65.7%)	142 (67.6%)		236 (76.9%)	59 (43.4%)		295 (66.6%)
Risk/benefit ratio	Agreement	164 (70.4%)	150 (71.4%)	0.950	259 (84.4%)	55 (40.4%)	<0.001	314 (70.9%)
	Disagreement	40 (17.2%)	36 (17.1%)		25 (8.1%)	51 (37.5%)		76 (17.2%)
Self-prioritization	Agreement	175 (75.4%)	150 (71.4%)	0.456	270 (88.2%)	55 (40.4%)	<0.001	325 (73.5%)
	Disagreement	46 (19.8%)	47 (22.4%)		24 (7.8%)	69 (50.7%)		93 (21%)
Global vaccine justice	Agreement	73 (31.3%)	68 (32.4%)	0.591	115 (37.5%)	26 (19.1%)	<0.001	141 (31.8%)
	Disagreement	67 (28.8%)	71 (33.8%)		64 (20.8%)	74 (54.4%)		138 (31.2%)
National vaccine justice	Agreement	78 (33.5%)	83 (39.5%)	0.650	128 (41.7%)	33 (24.3%)	<0.001	161 (36.3%)
	Disagreement	71 (30.5%)	68 (32.4%)		71 (23.1%)	68 (50%)		139 (31.4%)

*Mann-Whitney test (U) had been used with a significance level (Sig.) <0.05*.

*Bold values - statistically significant with p <0.05*.

### Determinants of COVID-19 VBD-related attitudes vs. uptake

On evaluating the demographic and anamnestic determinants of COVID-19 VBD-related attitudes, no significant difference was found among genders, age groups, pregnancy statuses, COVID-19 infection onset, COVID-19 infection severity, or vaccine type. The participants who had been previously infected by SARS-CoV-2 were significantly more inclined to reject the VBD (24.1 vs. 14.6%) and less inclined to accept the VBD (66 vs. 78.5%) than their counterparts. Contrarily, the participants who had been previously vaccinated against SARS-CoV-2 were significantly less inclined to reject the VBD (12.5 vs. 92.9%) and more inclined to accept the VBD (79.3 vs. 3.6%) than their counterparts. Hospital admission (35.7 vs. 11.7%) and seeking medical care (33.3 vs. 11.6%) were significantly associated with higher levels of COVID-19 VBD rejection (Table 5).

**Table 5:** Demographic and anamnestic determinants of COVID-19 vaccine booster acceptance among polish healthcare professionals and students responding to COVID-19 VBH survey, December 2021–January 2022 (*n* = 443).

**Variable**	**Outcome**	**Rejection (*****n*** = **78)**	***Sig***.	**Hesitancy (*****n*** = **35)**	***Sig***.	**Acceptance (*****n*** = **330)**	***Sig***.
Gender	Female^*^	57 (16.9%)	*Reference*	28 (8.3%)	*Reference*	253 (74.9%)	*Reference*
	Male	20 (19.8%)	0.496	6 (5.9%)	0.442	75 (74.3%)	0.904
	Diverse-gender	1 (25%)	0.670	1 (25%)	0.265	2 (50%)	0.279
Pregnancy^*^	Yes	1 (14.3%)	1.000	1 (14.3%)	0.457	5 (71.4%)	1.000
	No	56 (16.9%)		27 (8.2%)		248 (74.9%)	
Age group	>30 years-old	33 (18.6%)	0.579	16 (9%)	0.476	128 (72.3%)	0.354
	≤ 30 years-old	44 (16.6%)		19 (7.2%)		202 (76.2%)	
Prior COVID-19 infection	Yes^†^	34 (24.1%)	0.014	14 (9.9%)	0.280	93 (66%)	0.005
	No	44 (14.6%)		21 (7%)		237 (78.5%)	
Onset^†^	Before 1st dose	29 (28.2%)	*Reference*	8 (7.8%)	*Reference*	66 (64.1%)	*Reference*
	Between 1/2 doses	2 (33.3%)	0.785	0 (0%)	0.993	4 (66.7%)	0.898
	After 2nd dose	3 (9.4%)	0.039	6 (18.8%)	0.084	23 (71.9%)	0.418
Severity^†^	Asymptomatic	2 (50%)	*Reference*	1 (25%)	*Reference*	1 (25%)	*Reference*
	Mild	19 (20.4%)	0.188	8 (8.6%)	0.297	66 (71%)	0.091
	Moderate	13 (31.7%)	0.467	5 (12.2%)	0.483	23 (56.1%)	0.262
	Severe	0 (0%)	0.990	0 (0%)	0.991	3 (100%)	0.990
COVID-19 vaccination	Yes ^‡^	52 (12.5%)	<0.001	34 (8.2%)	0.714	329 (79.3%)	<0.001
	No	26 (92.9%)		1 (3.6%)		1 (3.6%)	
Vaccine type^^‡^^	Pfizer-BioNTech	38 (11.7%)	0.327	27 (8.3%)	0.871	260 (80%)	0.490
	Moderna	1 (5%)	0.491	2 (10%)	0.674	17 (85%)	0.777
	AstraZeneca-Oxford	5 (9.3%)	0.436	3 (5.6%)	0.599	46 (85.2%)	0.251
	Janssen	8 (50%)	<0.001	2 (12.5%)	0.631	6 (37.5%)	<0.001
Number of doses^^‡^^	One dose	8 (44.4%)	<0.001	5 (27.8%)	0.011	5 (27.8%)	<0.001
	Two doses	19 (21.1%)	0.005	17 (18.9%)	<0.001	54 (60%)	<0.001
	Three doses	25 (8.1%)	<0.001	12 (3.9%)	<0.001	270 (87.9%)	<0.001
Hospital admission^^‡^^	Yes	5 (35.7%)	0.021	2 (14.3%)	0.321	7 (50%)	0.013
	No	47 (11.7%)		32 (8%)		322 (80.3%)	
Medical care^^‡^^	Yes	6 (33.3%)	0.016	3 (16.7%)	0.175	9 (50%)	0.005
	No	46 (11.6%)		31 (7.8%)		320 (80.6%)	

*Logistic regression, Chi-squared test (χ^2^) and Fisher's exact test had been used with a significance level (Sig.) <0.05*.

* * Refers to female participants*.

† * Refers to the previously infected participants*.

‡ * Refers to the previously vaccinated participants*.

*Bold values - statistically significant with p <0.05*.

On evaluating the demographic and anamnestic determinants of COVID-19 VBD actual uptake, no significant difference was found among genders, age groups, pregnancy statuses, COVID-19 infection onset, COVID-19 infection severity, post-vaccination hospitalization, or seeking medical care. The participants who had been previously infected by SARS-CoV-2 had a significantly (*Sig*. <0.001) lower uptake level (58.2%) than their counterparts (74.5%). The participants who had been previously vaccinated against SARS-CoV-2 using Pfizer-BioNTech (80%) and Moderna (80%) had higher levels of VBD uptake than those who received AstraZeneca-Oxford (57.4%) and Janssen (0%) (Table 6).

**Table 6:** Demographic and anamnestic determinants of COVID-19 vaccine booster uptake among polish healthcare professionals and students responding to COVID-19 VBH survey, December 2021–January 2022 (*n* = 443).

**Variable**	**Outcome**	**Did not receive COVID-19 BD** **(*****n*** = **136)**	**Received COVID-19 BD** **(*****n*** = **307)**	***Sig***.
Gender	Female^*^	100 (29.6%)	238 (70.4%)	*Reference*
	Male	36 (35.6%)	65 (64.4%)	0.249
	Diverse-gender	0 (0%)	4 (100%)	0.984
Pregnancy^*^	Yes	4 (57.1%)	3 (24.9%)	0.202
	No	96 (29%)	235 (71%)	
Age group	>30 years-old	88 (33.2%)	177 (66.8%)	0.174
	≤ 30 years-old	48 (27.1%)	129 (72.9%)	
Prior COVID-19 infection	Yes^†^	59 (41.8%)	82 (58.2%)	<0.001
	No	77 (25.5%)	225 (74.5%)	
Onset^†^	Before 1st dose	40 (38.8%)	63 (61.2%)	*Reference*
	Between 1/2 doses	4 (66.7%)	2 (33.3%)	0.197
	After 2nd dose	15 (46.9%)	17 (53.1%)	0.420
Severity^†^	Asymptomatic	3 (75%)	1 (25%)	*Reference*
	Mild	38 (40.9%)	55 (59.1%)	0.211
	Moderate	17 (41.5%)	24 (58.5%)	0.228
	Severe	1 (33.3%)	2 (66.7%)	0.287
Vaccine type	Pfizer-BioNTech	65 (20%)	260 (80%)	<0.001
	Moderna	4 (20%)	16 (80%)	0.529
	AstraZeneca-Oxford	23 (42.6%)	31 (57.4%)	0.003
	Janssen	16 (100%)	0 (0%)	<0.001
Hospital admission	Yes	7 (50%)	7 (50%)	0.058
	No	101 (25.2%)	300 (74.8%)	
Medical care	Yes	8 (44.4%)	10 (55.6%)	0.095
	No	100 (25.2%)	297 (74.8%)	

*Logistic regression, Chi-squared test (χ^2^) and Fisher's exact test had been used with a significance level (Sig.) <0.05*.

* * Refers to female participants*.

† * Refers to the previously infected participants*.

‡ * Refers to the previously vaccinated participants*.

*Bold values - statistically significant with p <0.05*.

The bivariate correlation between COVID-19 VBD-related attitudes and actual uptake revealed that there had been moderate and positive correlation between VBD-related acceptance and number of doses (ρ = 0.508; *Sig*. <0.001) and being triple vaccinated (ρ = 0.464; *Sig*. <0.001). Contrarily, there correlation was moderate and negative between VBD-related rejection and number of doses (ρ = −0.437; *Sig*. <0.001) and being triple vaccinated (ρ = −0.373; *Sig*. <0.001) (Table 7).

**Table 7:** Correlation between vaccine doses & willingness to receive COVID-19 vaccine booster doses.

		**Rejection**	**Hesitancy**	**Acceptance**
Number of dose	Spearman's ρ	−0.437	−0.204	0.508
	*Sig*.	<0.001	<0.001	<0.001
Triple vaccinated	Spearman's ρ	−0.373	−0.222	0.464
	*Sig*.	<0.001	<0.001	<0.001

*Bivariate correlation had been used with a significance level (Sig.) <0.05*.

*Bold values - statistically significant with p <0.05*.

### Regression analysis of COVID-19 VBD-related acceptance determinants

The multivariable logistic regression of psychosocial drivers of COVID-19 VBD-related acceptance was adjusted for prior infection, vaccine type, number of doses, hospitalization, and medical care. The participants who agreed with the severe infection notion had an increased adjusted odds ratio (AOR) of 5.142 (CI 95%: 2.346–11.269) times to accept COVID-19 VBD. Similarly, agreement with the symptomatic infection (AOR: 5.502; CI 95%: 2.717–11.139), community transmission (AOR: 5.898; CI 95%: 3.041–11.438), equal safety (AOR: 3.733; CI 95%: 1.622–8.592), favorable risk-benefit ratio (AOR: 9.573; CI 95%: 4.461–20.544), and self-prioritization (AOR: 17.407; CI 95%: 8.382–36.150) had an increased odd to accept COVID-19 VBD. On the other hand, agreement with the notion of variant control decreased the odds of accepting COVID-19 VBD (AOR: 0.143; CI 95%: 0.072–0.286). Ignoring the ethical dilemmas globally (AOR: 2.584; CI 95%: 1.274–5.242) and nationally (AOR: 2.426; CI 95%: 1.233–4.772) was associated with increased odds of VBD acceptance (Table 8).

**Table 8:** Psychosocial determinants of COVID-19 vaccine booster acceptance among polish healthcare professionals and students responding to COVID-19 VBH survey, December 2021–January 2022 (*n* = 443).

**Determinant**	**B (SE)**	**Wald**	**AOR**	**CI 95%**	***Sig***.
**Severe infection**: agree (vs. disagree)	1.637 (0.400)	16.728	5.142	2.346–11.269	<0.001
**Symptomatic infection**: agree (vs. disagree)	1.705 (0.360)	22.442	5.502	2.717–11.139	<0.001
**Community transmission**: agree (vs. disagree)	1.775 (0.338)	27.575	5.898	3.041–11.438	<0.001
**Variants control**: agree (vs. disagree)	−1.942 (0.352)	30.482	0.143	0.072–0.286	<0.001
**Equal safety**: agree (vs. disagree)	1.317 (0.425)	9.591	3.733	1.622–8.592	0.002
**Daily routine**: disagree (vs. agree)	0.461 (0.413)	1.245	1.585	0.706–3.563	0.265
**Risk/benefit ratio**: agree (vs. disagree)	2.259 (0.390)	33.618	9.573	4.461–20.544	<0.001
**Self-prioritization**: agree (vs. disagree)	2.857 (0.373)	58.706	17.407	8.382–36.150	<0.001
**Global vaccine justice**: agree (vs. disagree)	0.949 (0.361)	6.921	2.584	1.274–5.242	0.009
**National vaccine justice**: agree (vs. disagree)	0.886 (0.345)	6.589	2.426	1.233–4.772	0.010

*Binary logistic regression had been adjusted for prior infection, vaccine type, number of doses, hospitalization, and medical care with a significance level (Sig.) <0.05*.

*Bold values - statistically significant with p <0.05*.

### Regression analysis of COVID-19 VBD uptake determinants

The multivariable logistic regression of psychosocial drivers of COVID-19 VBD actual uptake was adjusted for prior infection and vaccine type. The participants who agreed with the severe infection notion had an increased adjusted odds ratio (AOR) of 4.283 (CI 95%: 2.051–8.941) times to receive COVID-19 VBD. Similarly, agreement with the symptomatic infection (AOR: 4.347; CI 95%: 2.284–8.275), community transmission (AOR: 4.179; CI 95%: 2.268–7.700), equal safety (AOR: 2.323; CI 95%: 1.024–5.273), favorable risk-benefit ratio (AOR: 3.589; CI 95%: 1.779–7.241), and self-prioritization (AOR: 6.984; CI 95%: 3.690–13.216) had an increased odd to receive COVID-19 VBD. On the other hand, agreement with the notion of variants control decreased the odds of receiving COVID-19 VBD (AOR: 0.169; CI 95%: 0.091–0.314). Ignoring the ethical dilemmas globally (AOR: 2.501; CI 95%: 1.360–4.600) and nationally (AOR: 1.819; CI 95%: 1.012–3.269) was associated with increased odds of VBD acceptance (Table 9).

**Table 9:** Psychosocial determinants of COVID-19 vaccine booster uptake among polish healthcare professionals and students responding to COVID-19 VBH survey, December 2021–January 2022 (*n* = 443).

**Determinant**	**B (SE)**	**Wald**	**AOR**	**CI 95%**	***Sig*.**
**Severe infection**: agree (vs. disagree)	1.455 (0.376)	15.002	4.283	2.051–8.941	<0.001
**Symptomatic infection**: agree (vs. disagree)	1.470 (0.328)	20.016	4.347	2.284–8.275	<0.001
**Community transmission**: agree (vs. disagree)	1.430 (0.312)	21.037	4.179	2.268–7.700	<0.001
**Variants control**: agree (vs. disagree)	−1.780 (0.317)	31.578	0.169	0.091–0.314	<0.001
**Equal safety**: agree (vs. disagree)	0.843 (0.418)	4.063	2.323	1.024–5.273	0.044
**Daily routine**: disagree (vs. agree)	−0.693 (0.404)	2.946	0.500	0.227–1.103	0.086
**Risk/benefit ratio**: agree (vs. disagree)	1.278 (0.358)	12.732	3.589	1.779–7.241	<0.001
**Self-prioritization**: agree (vs. disagree)	1.944 (0.325)	35.664	6.984	3.690–13.216	<0.001
**Global vaccine justice**: agree (vs. disagree)	0.917 (0.311)	8.699	2.501	1.360–4.600	0.003
**National vaccine justice**: agree (vs. disagree)	0.598 (0.299)	3.998	1.819	1.012–3.269	0.046

*Binary logistic regression had been adjusted for prior infection and vaccine type with a significance level (Sig.) <0.05*.

*Bold values - statistically significant with p <0.05*.

## Discussion

Vaccine acceptance is perceived essential to curb the COVID-19 pandemic. The present cross-sectional study involved Polish HCPs and MUSs to understand the drivers of VBH among this particular population subset. Our findings revealed that almost three-quarters (74.5%) of the participants favored receiving the COVID-19 VBD, while 17.6 and 7.9% indicated their rejection and uncertainty, respectively. These results are consistent with the previously published studies by Rzymski et al. (13) and Babicki and Mastalerz-Migas (14), who found that about 71 and 70% of Polish adults were interested in receiving COVID-19 VBD as soon as possible. Likewise, the studies in other high-income countries, such as the Czech Republic (71.3%), Germany (87.8%), Italy (85.7%), Japan (97.9%), Singapore (73.8%), and the United States (92.2%), exhibited high levels of COVID-19 VBD acceptance, especially among HCPs (21, 26, 31–34). On the other hand, the studies in low- and middle-income countries such as Algeria (51.6%), China (60.1%), and Jordan (44.6%) exhibited lower acceptance levels, especially among non-HCPs groups (35–37). A suggested explanation for intra- and inter-country variance in VBH levels is the respondents' health literacy level which is supposed to be higher among HCPs compared with other population subsets; therefore, the study among adult Americans by Yadete et al. (38) found lower acceptance for COVID-19 VBD (62%) than what Pal et al. (33) reported for American HCPs (92.2%). Similarly, Babicki and Mastalerz-Migas (14) found significant differences in COVID-19 VBD acceptance between Polish HCPs and non-HCPs. It is irrefutable that elements of the health belief model such as perceived susceptibility, perceived benefits, and perceived barriers contribute to this significant difference between HCPs and other groups; therefore, the goal of this study was to explore VBH drivers among HCPs, including the psychosocial benefits and barriers (39–41).

Regarding the representativeness of our sample, the latest figures published by the EU Labor Force Survey in 2021 revealed that 82.5% of Polish HCPs were females, thus justifying the female predominance of our sample (75.1%) (42). Similarly, the Organization for Economic Co-operation and Development (OECD) revealed that about 75% of Polish students enrolled in health and welfare-related programs were females, which is similar to our female students' proportion (77.6%) (43). The median age of the Polish population was 41.7 years in the year 2020, while the mean age of the sample was 31.1 ± 11.4 years, with a statistically significant difference (*Sig*. <0.001) between professionals (38.8 ± 10.9 years) and students (22.6 ± 2.3 years) (44). According to the Public Opinion Research Center (CBOS; Warsaw, Poland) report of 2021, about 61% of the fully vaccinated Polish citizens, i.e. those who received two primer doses, received Pfizer-BioNTech, while 22% received AstraZeneca-Oxford, 12% received Moderna, and only 3% received Janssen (45). Interestingly, Pfizer-BioNTech was the most administered vaccine among our participants who received primer doses only (60.2%), followed by AstraZeneca-Oxford (21.3%), Janssen (14.8%), and Moderna (3.7%). It is worthy to note that Pfizer-BioNTech was significantly (*Sig*. <0.001) more common among HCPs (89.3%) than MUSc (66.7%), while AstraZeneca-Oxford were significantly (*Sig*. <0.001) more common among MUSc (22.9%) than HCPs (3.7%). The decision to prioritize HCPs for receiving COVID-19 vaccines in early 2021 in Poland can explain this significant difference between HCPs and MUSs in terms of vaccines types, as the authorization of Pfizer-BioNTech was earlier and the number of its purchased doses was higher than other COVID-19 vaccine brands (46).

Around one-third (32%) of our participants had a prior COVID-19 infection, with a different severity. As per the WHO data, by April 14, 2022, 5.9 million total COVID-19 cases were reported in Poland, representing 15.5% of the total population, with a total of 54,165,921 vaccine doses have been administered by April 10, 2022 (47). This difference could be attributed to the inclusion of only HCPs and MUSc in our study. In most participants (73%), COVID-19 infections occurred before the vaccination, while around 23% of cases occurred after the second dose of the vaccine. Similarly, Klugar et al. (21) found that around 90.9% of COVID-19 infections occurred before the first dose, while only 7.3% after the second dose among Czech HCPs.

The most common reason influencing VBD acceptance among our participants was the protection of one's health (96.3%), followed by protection of family's health (82.5%), community's health (65%) and patients' or colleagues' health (56.7%). Similarly, Attia et al. (26) found that among German university staff and students, the most commonly reported promoter was the protection of one's health (95.6%), followed by the protection of the community's health (91.6%) and family's health (91.2%). In the Czech Republic, protection of family's health (83%) was the most commonly reported promoter, followed by protection of one's health (82.7%), patients' or colleagues' health (70.4%) and community's health (66.4%) (21). Even for primer doses, the HCPs' most frequently reported reason for accepting them in the United States was the protection of family's health (86.7%), followed by protection of one's health (82.9%), and community's health (68.8%) (48). In Palestine, COVID-19 vaccine acceptance was substantially higher among the nurses who were more concerned about protecting their families and patients (49). Likewise, Szmyd et al. (50) revealed that the most commonly reported COVID-19-related concern among Polish HCPs was health deterioration in family members (70.3%) which was significantly (*Sig*. <0.001) more common than Polish non-HCPs (55.9%). Moreover, Szmyd et al. (50) found that the physicians' family members (67.5%) were reportedly (*Sig*. <0.001) more infected by SARS-CoV-2 than non-HCPs' family members (54.7%).

About 13.8% of our participants disagreed with the statement that COVID-19 VBD will be as safe as the primer doses, with a considerable difference between those who were triple-vaccinated (6.2%) and non-tripled vaccinated (30.9%); thus, indicating the role of post-vaccination safety and side effects in determining the attitudes toward COVID-19 VBD. Al-Qerem et al. (37) found that fear of severe side effects following COVID-19 VBD (34.1%) and the incapacity to tolerate primer doses side effects (24.6%) were the most commonly reported reasons for COVID-19 VBD rejection among Jordanian adults. Likewise, post-vaccination side effects were main reasons for COVID-19 VBH in Algeria (35). Heretofore all authorized COVID-19 vaccines have been proven safe since phase II/III trials conducted by manufacturers (51). Therefore, the continuation of phase IV studies conducted by independent institutions and regulators is vital to protect the public confidence in vaccines (51–55).

The participants who had been previously infected by SARS-CoV-2 were significantly more inclined to reject the VBD, whereas the participants who had been previously vaccinated against SARS-CoV-2 were more willing to accept the VBD. A Lebanese web-based cross-sectional study using the health belief model also supported the notion that HCPs who had been previously diagnosed with COVID-19 were significantly associated with a lower level of vaccine acceptance (56). The misconception of natural immunity triggered by prior infection can explain this finding, and it had been one of the key drivers for vaccine hesitancy proposed by the WHO-SAGE (56, 57).

The applied comprehensive multivariable logistic regression model for the psychosocial drivers of COVID-19 VBD-related acceptance and uptake revealed that the participants who agreed with severe infection, symptomatic infection, and community transmission notions had higher odds of accepting. The effectiveness of vaccines, especially VBD, was a primary promoter for COVID-19 VBD-related acceptance among Algerian adults, American adults, German university students and staff, and Italian university students (26, 34, 35, 38). Using the ministry of health database, a nationwide population-based study from Israel found that COVID-19 VBD reduced the risk of developing COVID-19 infection and severe illness among VBD recipients (58). In our study, effectiveness against the emerging variants was a prominent determinant for VBD-related acceptance and uptake, consistent with what was found earlier among Czech HCPs and German university students and staff (21, 26).

The risk-benefit profile of VBD impacted COVID-19 booster dose acceptance because a positive association between the COVID-19 VBD acceptance and perceived susceptibility, as well as benefit. Public health campaigns are expected to highlight the postulated benefits of vaccines, especially in terms of effectiveness against symptomatic and severe infection, along with the expected harms of unvaccinated population (26, 59).

### Strengths

The present study is the first to particularly target HCPs and MUSs in Poland. Participants' identity was kept confidential and anonymous to control Hawthorne's effect. The crucial findings may help promote the booster dose uptake worldwide.

### Limitations

The non-random sampling technique used to recruite participants of this study may partially limited the representativeness of obtained results. HCPs and MUSc are much more aware than the general population in terms of the risk-benefit profile of vaccines, and they are more prone to show high vaccine uptake and acceptance; Hence, this study's findings should not be directly applied to the general population. The non-random sampling approach used might be linked with selection bias; whereas, the sample was relatively representative considering metropolitan areas of vast majority of participants. Some professional groups were disproportionally represented in our sample, as a few of their members participated in this study; therefore, future studies on HCPs should aim for representing professional groups proportionately. In addition, online surveys could contribute to measurement bias as fraction of participants tend not to fully respond to the all questionnaire items. Our findings will support a rationale for efficient dissemination of booster doses of COVID-19 vaccines.

## Conclusion

A high vaccine acceptance among HCPs and MUSc in Poland indicate the positive attitude of these groups toward mass inoculation. The previous infection by SARS-CoV-2 significantly increased a risk of VBD hesitancy. Protection from severe infection, community transmission, good safety profile, and favorable risk-benefit ratio were the significant determinants of the COVID-19 VBD acceptance and uptake. The enhanced public health campaigns are designed to highlight the postulated benefits of vaccines and the expected harms of skipping VBD.

## Data availability statement

The raw data supporting the conclusions of this article will be made available by the authors, without undue reservation.

## Ethics statement

The studies involving human participants were reviewed and approved by Ethics Committee of the Medical University of Silesia. The patients/participants provided their written informed consent to participate in this study.

## Author contributions

AR: conceptualization, methodology, and formal analysis. AD: software and funding acquisition. JI and AD: validation. AD, JI, and MK: investigation. AR, JI, and SH: writing—original draft preparation. AD, MT, RW, and RK: writing—review and editing. AR and AD: supervision and project administration. All authors have read and agreed to the published version of the manuscript.

## Funding

The work of AR was supported by Masaryk University grants numbers MUNI/IGA/1104/2021 and MUNI/A/1402/2021. JI was a participant of the STER Internationalization of Doctoral Schools Program from NAWA Polish National Agency for Academic Exchange No. PPI/STE/2020/1/00014/DEC/02. The work of SH was supported by Operational Programme Research, Development and Education—Project, Postdoc2MUNI (No. CZ.02.2.69/0.0/0.0/18_053/0016952). The work of AD was supported by grant of Medical University of Silesia: PCN-1-203/K/1/I and PCN-176/K/0/I.

## Conflict of interest

The authors declare that the research was conducted in the absence of any commercial or financial relationships that could be construed as a potential conflict of interest.

## Publisher's note

All claims expressed in this article are solely those of the authors and do not necessarily represent those of their affiliated organizations, or those of the publisher, the editors and the reviewers. Any product that may be evaluated in this article, or claim that may be made by its manufacturer, is not guaranteed or endorsed by the publisher.
